# Detection of QTLs for Yield Heterosis in Rice Using a RIL Population and Its Testcross Population

**DOI:** 10.1155/2016/2587823

**Published:** 2016-12-22

**Authors:** Yu-Jun Zhu, De-Run Huang, Ye-Yang Fan, Zhen-Hua Zhang, Jie-Zheng Ying, Jie-Yun Zhuang

**Affiliations:** State Key Laboratory of Rice Biology and Chinese National Center for Rice Improvement, China National Rice Research Institute, Hangzhou 310006, China

## Abstract

Analysis of the genetic basis of yield heterosis in rice was conducted by quantitative trait locus mapping using a set of 204 recombinant inbred lines (RILs), its testcross population, and mid-parent heterosis dataset (H_MP_). A total of 39 QTLs for six yield traits were detected, of which three were detected in all the datasets, ten were common to the RIL and testcross populations, six were common to the testcross and H_MP_, and 17, 2, and 1 were detected for RILs, testcrosses, and H_MP_, respectively. When a QTL was detected in both the RIL and testcross populations, the difference between TQ and IR24 and that between Zh9A/TQ and Zh9A/IR24 were always in the same direction, providing the potential to increase the yield of hybrids by increasing the yield of parental lines. Genetic action mode of the 39 QTLs was inferred by comparing their performances in RILs, testcrosses, and H_MP_. The genetic modes were additive for 17 QTLs, dominance for 12 QTLs, and overdominance for 10 QTLs. These results suggest that dominance and overdominance are the most important contributor to yield heterosis in rice, in which the accumulative effects of yield components play an important role.

## 1. Introduction

Heterosis, or hybrid vigor, refers to the superior performance of hybrids relative to their parents. It plays an important role for enhancing crop yield. Rice is the staple food crop feeding over half of the world's population. Currently, rice hybrids are widely adopted in many countries, especially in China where hybrid rice varieties occupy 57% of the rice-growing area [1]. In the last two decades, quantitative trait locus (QTL) mapping has become a major approach to characterize the contribution of individual genomic regions to heterosis. A total of 17 crosses have been used, including eight* indica* ×* indica* crosses [2–9], seven* indica* ×* japonica* crosses [10–16], one* japonica* ×* japonica* cross [17], and one cross between* Oryza rufipogon* Griff. and* O. sativa* L. [18]. In addition, different types of segregating populations derived from the same cross have been applied, for example, the* F*_2:3_ families [2] and immortalized* F*_2_ populations [8] derived from Zhenshan 97/Minghui 63 and recombinant inbred lines (RILs) derived from Lemont/Teqing and its backcross and testcross populations [11]. Based on single and two-locus QTL analysis, dominance [10], overdominance [3, 14, 15, 18, 19], epistasis [2, 16, 20], and accumulated effects [8, 12, 21–25] could be the major determinants of heterosis in rice. With the development of second-generation genome sequencing technologies, genome-wide association studies have become a new effective approach to elucidate the genetic bases of complex traits in rice. Using 1,495 elite hybrid rice varieties and their inbred parental lines, Huang et al. reported that accumulation of superior alleles with positive dominance is an important contributor to the heterotic phenomena [26].

Testcrossing is the most common way to identify superior hybrids in plant breeding. Consequently, testcross populations have been widely used to identify QTLs associated with yield heterosis in rice. Using populations produced by crossing RILs of Lemont/Teqing to the two parental lines and two testers (Zhong 413 and IR64), Li et al. [11] and Luo et al. [19] found that epistasis and overdominance were important for heterosis. Using four testcross populations derived from a set of RILs, You et al. [5] found that QTLs having the strongest effect for each of the seven traits in the RILs were detected in two or more testcross populations across different environments, thus suggesting that these QTLs are important for hybrid rice breeding. Xiang et al. [9] found that 56 of the 62 QTLs detected had significant effects in at least two of the four testcross populations and suggested that accumulation of various components of the QTL effects may adequately explain the genetic basis of heterosis.

In the present study, we developed a testcross population by crossing 204 RILs derived from two elite restorer lines with a male sterile line that was commonly used in hybrid rice breeding [27]. This study is aiming to detect and evaluate QTLs controlling grain yield and its component traits using datasets of the RILs, testcross* F*_1_s, and the mid-parent heterosis and to analyze the genetic basis of heterosis in rice.

## 2. Materials and Methods

### 2.1. Plant Materials

One RIL population consisting of 204 lines and a testcross population produced by crossing the RILs to the cytoplasmic male sterile line Zhong 9A (Zh9A) were used in this study. The RIL population was constructed and used previously [28], in which the female parent Teqing (TQ) is an* indica* inbred variety and restorer line of the three-line hybrid rice, and the male parent includes a number of near isogenic lines in the genetic background of the restorer line IR24.

### 2.2. Phenotypic Evaluation

The two populations, as well as TQ, IRBB52, IRBB59, Zhong 9B (Zh9B), and the* F*_1_ hybrids between Zh9A and TQ, IRBB52 and IRBB59, were planted at the China National Rice Research Institute, Zhejiang, China. They were tested in 2009 and 2011 with sowing on May 27 and May 24 and transplanting on June 20 and June 17, respectively. The experiments followed a randomized complete block design with two replications. The planting density was 16.7 cm between plants and 26.7 cm between rows. Each pair of a testcross* F*_1_ and the RIL used as its male parent were planted side by side. Zh9B was planted at intervals of every 20 rows in the RIL and testcross populations, respectively. Field management followed the normal agricultural practice. At maturity, the middle four plants of each replication for each line were harvested together. Six yield traits, including number of panicles per plant (NP), number of grains per panicle (NGP), number of spikelets per panicle (NSP), spikelet fertility (SF), 1000-grain weight (TGW), and grain yield per plant (GY) were measured.

### 2.3. Data Analysis

The mid-parent heterosis (H_MP_) was calculated as H_MP_ =* F*_1_ − MP, where* F*_1_ is the trait value of a testcross* F*_1_ and MP is the mean value of the corresponding paternal RIL and the common maternal line Zh9B. SAS Proc GML [29] was used to test the differences between the RILs and the testcross* F*_1_s. Broad-sense heritability (*H*) was estimated using the following formula:* H* (%) = 100 × *V*_*G*_/*V*_*P*_, in which *V*_*G*_ and *V*_*P*_ are the genotypic and phenotypic variances, respectively.

The linkage map was constructed previously, which consisted of 127 markers including two STSs and 125 SSRs. It spanned 1197.7 cM with larger gaps remaining on chromosomes 1 and 4 [28]. Main-effect QTLs and genotype-by-environment (GE) interactions were determined using QTL Network 2.0 [30], in which the year was treated as an environment factor. Genome-wise type I errors were calculated with 1000 permutation test. A threshold of *P* < 0.05 was used for detecting candidate QTLs and significant QTL regions, while a threshold of *P* < 0.01 was used for claiming a significant QTL effect. The proportion of phenotypic variance explained by a QTL and GE interaction (*R*^2^), as well as the overall *R*^2^ jointly explained by all the QTLs or GE interactions detected for a given trait in a given population, were calculated, respectively. In the genome scan, testing window of 10 cM, filtration window of 10 cM, and walk speed of 1 cM were chosen. QTLs were designated following the rules recommended by McCouch and CGSNL [31]. Genetic parameters of the QTL effects detected using the three sets of data are showed in Table 1. The QTL effects detected in the testcross population indicate differences between the two types of heterozygotes, Zh9A/TQ and Zh9A/IR24, which is equivalent to the standard heterosis used in the breeding practice [32]. The QTL effects detected using H_MP_ dataset indicate the dominance component of the standard heterosis. Thus, all the QTLs detected using the testcross and H_MP_ datasets are genetic loci underlying standard heterosis in rice.

## 3. Result

### 3.1. Phenotypic Performances

Mean trait values of the parent lines, reference* F*_1_s, and segregating populations are shown in Table 2. The three reference* F*_1_s, Zh9A/TQ, Zh9A/IRBB52, and Zh9A/IRBB59, had higher values for all the traits than the common female parent Zh9B. As compared with TQ, Zh9A/TQ showed higher values for NP and GY, similar values for NSP and TGW, and lower values for NGP and SF. As compared with IRBB52 and IRBB59, Zh9A/IRBB52 and Zh9A/IRBB59 showed higher values for NGP, NSP, TGW, and GY, and lower values for NP and SF, respectively. Comparison between mean trait values of the RIL and testcross populations showed that the two populations had no significant difference on NP (*P* = 0.6275) and NGP (*P* = 0.1399), while the values in the testcross population were higher on NSP (*P* < 0.0001), TGW (*P* = 0.0011), and GY (*P* = 0.0040) but lower on SF (*P* < 0.0001) than in the RIL population. These results indicated that yield heterosis was produced by the interaction between genes in the male sterile line Zh9A and the restorer lines TQ, IRBB52, and IRBB59, but the effects may be weakened due to low seed setting rate.

**Figure 1:**
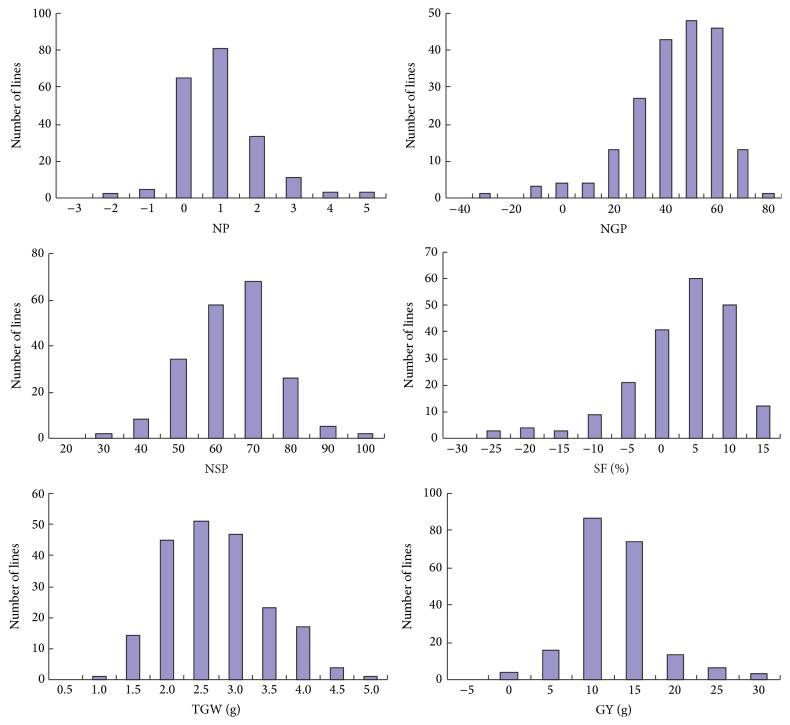
Distribution of the mid-parent heterosis values for the six yield traits. NP, number of panicles per plant; NGP, number of grains per panicle; NSP, number of spikelets per panicle; SF, spikelet fertility; TGW, 1000-grain weight; GY, grain yield per plant.

For H_MP_ that is a derivative parameter measuring the mid-parent heterosis, the direction and magnitude varied greatly among different yield traits (Figure 1). For NSP and TGW, all the testcross* F*_1_s showed positive H_MP_ values. For NGP and GY, only eight and four* F*_1_s did not have positive values, respectively. For NP and SF, 72 and 81* F*_1_s showed negative values, respectively. These results indicated that heterosis in grain yield was presented in the testcrosses which was mainly ascribed to grain weight and grain number.

### 3.2. Correlation between the Performances of RILs, Testcross* F*_1_s, and Mid-Parent Heterosis

Correlation coefficients between the performances of RILs, testcross* F*_1_s, and *H*_MP_ are shown in Table 3. Significant positive correlations (*P* < 0.01) between the RILs and testcross* F*_1_s were observed for all the traits. The correlation coefficient was the highest for TGW (0.902), followed from high to low by NSP (0.675), NGP (0.446), SF (0.406), GY (0.377), and NP (0.375). For *H*_MP_ that was determined by the trait values of the testcross* F*_1_s and RILs, its correlations with testcross* F*_1_s and RILs were highly different. The correlation coefficients between testcross* F*_1_s and H_MP_ were significant (*P* < 0.01) and positive for all the traits. The correlation coefficient was the highest for SF (0.953), followed from high to low by GY (0.925), NP (0.882), NGP (0.840), NSP (0.684), and TGW (0.598). On the contrarily, the correlations between RILs and H_MP_ were low for all of the traits although significant correlation was detected for one of the traits.

### 3.3. Heritability of the Six Yield Traits in the RIL and Testcross Populations

Broad-sense heritability of the yield traits in the RIL and testcross populations is presented in Table 4. In the RIL population, the highest heritability of 94.50% was detected for TGW, which was much higher than the values of 26.64–54.92% for other traits. In the testcross population, the highest heritability of 84.93% was also detected for TGW. Again this value was considerably higher than the values 12.27–66.17% for other traits. These results indicated that the variation of TGW was largely contributed by the genetic effect and the remaining five traits were easily affected by environmental factors.

### 3.4. QTL Detected for the Six Yield Traits

**Figure 2:**
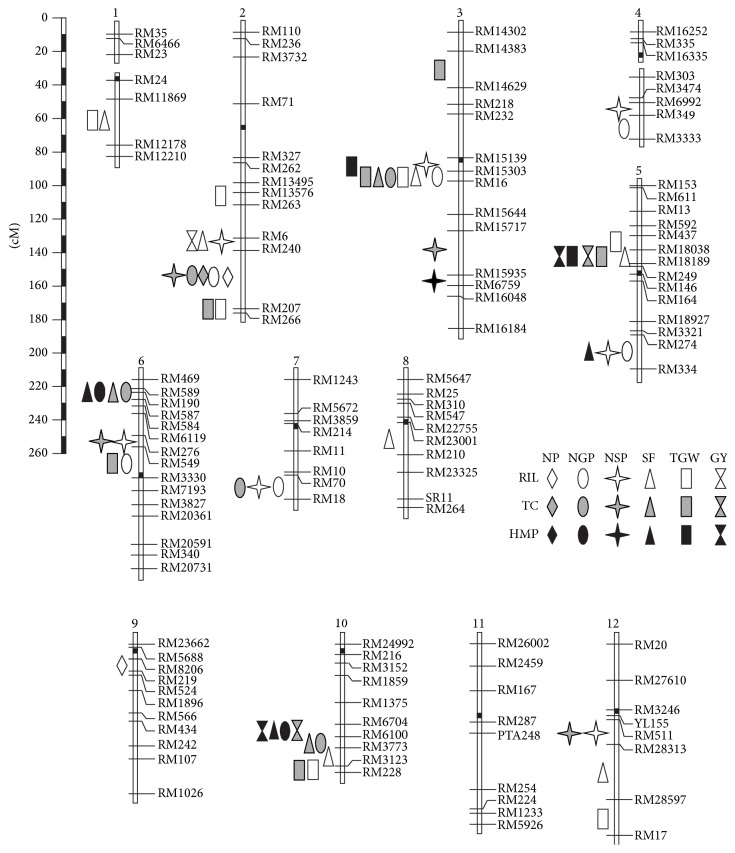
Chromosomal (numbered on the top) location of QTLs for the six yield traits. NP, number of panicles per plant; NGP, number of grains per panicle; NSP, number of spikelets per panicle; SF, spikelet fertility; TGW, 1000-grain weight; GY, grain yield per plant; RIL, recombinant inbred line; TC, testcross* F*_1_; *H*_MP_, mid-parent heterosis.

A total of 30, 21, and 10 QTLs were detected for the RILs, testcrosses, and H_MP_, with the phenotypic variance explained by a single QTL ranging as 1.01–27.21%, 0.52–37.83%, and 0.99 to 23.01%, respectively (Table 5). They distributed on all the 12 rice chromosomes except chromosome 11 (Figure 2).

Two QTLs for NP were detected, including* qNP2* detected in both the RIL and testcross populations and* qNP9* detected in the RIL population only. At* qNP2*, the IR24 allele increased NP by 0.66 compared with the TQ allele and the Zh9A/IR24 heterozygote raised NP by 0.94 compared with the Zh9A/TQ heterozygote, having *R*^2^of 9.49 and 4.98%, respectively. At* qNP9*, the IR24 allele increased NP by 0.35, having *R*^2^ of 4.06%. The two QTLs jointly explained 13.55% phenotypic variance in the RIL population. None of these QTLs showed significant GE interaction.

Eight QTLs for NGP were detected. Three QTLs,* qNGP2*,* qNGP3,* and* qNGP7*, were detected in both the RIL and testcross populations, with the TQ allele increasing NGP by 13.06, 6.68, and 5.54, and the Zh9A/TQ heterozygote increased NGP by 12.17, 10.32, and 10.99, respectively. Two QTLs,* qNGP6.1 *and* qNGP10*, were common to testcross* F*_1_s and H_MP_, with the Zh9A/IR24 heterozygote increasing NGP by 7.31 and 18.88 for testcross* F*_1_s and 8.30 and 22.31 for H_MP_, respectively. The remaining three QTLs were only detected in RILs, with the IR24 allele increasing NGP at* qNGP4 *and* qNGP5* but decreasing NGP at* qNGP6.2*. Among these QTLs,* qNGP3* was the only one showing a significant GE effect. Overall *R*^2^ of the QTLs detected for the RILs, testcrosses, and H_MP_ were 15.59, 13.48, and 25.50%, respectively.

Eight QTLs for NSP were detected, none of which had significant GE interaction. Three QTLs,* qNSP2*,* qNSP6,* and* qNSP12,* were detected in both the RIL and testcross populations. At* qNSP2* and* qNSP6*, the TQ allele increased NSP by 14.06 and 7.47, and the Zh9A/TQ heterozygote increased NSP by 19.16 and 9.81, respectively. At* qNSP12*, the TQ allele and the Zh9A/TQ heterozygote decreased NSP by 6.13 and 11.50, respectively. One QTL,* qNSP3.2*, was detected for both the testcross and H_MP_, with the Zh9A/TQ heterozygote increasing NSP by 7.62 and 7.19, respectively. Four QTLs,* qNSP3.1*,* qNSP4*,* qNSP5,* and* qNSP7*, were only detected in RILs. The IR24 allele increased NSP by 6.24 and 6.04 at* qNSP4* and* qNSP5* and decreased NSP by 5.46 and 6.55 at* qNSP3.1* and* qNSP7*, respectively. Overall *R*^2^ of the QTLs detected for the RILs, testcrosses, and H_MP_ were 15.41, 4.02, and 2.99%, respectively.

Nine QTLs for SF were detected. One QTL,* qSF10, *was detected in all the three datasets. While the IR24 allele increased SF by 1.04%, the Zh9A/IR24 heterozygote increased SF by 10.88 and 10.09% in testcrosses and H_MP_, respectively. One QTL,* qSF3*, was detected in both the RIL and testcross populations, with the TQ allele and Zh9A/TQ heterozygote increasing SF by 1.28 and 3.95%, respectively. One QTL,* qSF6*, was detected for both the testcrosses and H_MP_, with the IR24 allele and Zh9A/IR24 heterozygote increasing SF by 4.46 and 3.71%, respectively. One QTL,* qSF5.2*, was only detected for H_MP_, with the Zh9A/IR24 heterozygote increasing SF by 3.72%. The remaining five QTLs were only detected in RILs. The IR24 allele increased SF at* qSF2*,* qSF5.1,* and* qSF8* but decreased SF at* qSF1* and* qSF12*. One of them,* qSF5.1*, was the only QTL showing a significant GE effect for SF. Overall *R*^2^ of the QTLs detected for the RILs, testcrosses, and H_MP_ were 27.85, 32.35, and 25.09%, respectively.

Nine QTLs for TGW were detected, none of which had significant GE interaction. Two QTLs were detected in all the three datasets. The* qTGW3.2* had the largest *R*^2^ of 27.21, 37.83, and 6.65% in RILs, testcrosses, and H_MP_, respectively. While the IR24 allele increased TGW by 1.45 g, the Zh9A/IR24 heterozygote increased TGW by 2.33 g and 0.84 g in testcrosses and H_MP_, respectively. The* qTGW5* had the second largest *R*^2^ of 17.71% in RILs and much smaller *R*^2^ of 6.16 and 1.48% in testcrosses and H_MP_, respectively. While the IR24 allele decreased TGW by 1.25 g, the Zh9A/IR24 heterozygote decreased TGW by 0.90 g in testcrosses and increased TGW by 0.41 g in H_MP_. Two QTLs were detected in both the RIL and testcross populations. At* qTGW2.2*, the IR24 allele and Zh9A/IR24 heterozygote increased TGW by 0.36 and 0.61 g, respectively. At* qTGW10*, the IR24 allele and Zh9A/IR24 heterozygote decreased TGW by 0.32 and 0.51 g, respectively. Three QTLs,* qTGW1*,* qTGW2.1,* and* qTGW12,* were only detected in RILs, of which the enhancing alleles were all derived from IR24. The remaining two QTLs,* qTGW3.1* and* qTGW6*, were only detected in testcrosses, with the Zh9A/IR24 heterozygote decreasing and increasing TGW, respectively. Overall *R*^2^ of the QTLs detected for the RILs, testcrosses, and H_MP_ were 65.23, 58.55, and 8.12%, respectively.

Three QTLs for GY were detected. Two of them,* qGY5* and* qGY10,* were detected for both the testcrosses and H_MP_, with the Zh9A/IR24 heterozygote decreasing and increasing GY, respectively. The two QTLs jointly explained 8.99 and 8.80% of the phenotypic variance in testcrosses and H_MP_, respectively. The other QTL,* qGY2*, was only detected in RILs, having *R*^2^ of 3.63% with the TQ allele increasing GY by 1.17 g. This QTL also had a significant GE effect.

## 4. Discussion

QTL mapping has greatly enriched our understanding in the genetic basis of heterosis in rice. The mapping was taken in the present study by using testcross progenies derived from crosses between breeding lines with the cytoplasmic male sterile line Zh9A. The parental lines TQ and IR24 of the RIL population are two important inbred varieties and restorer lines used in China [33], and Zh9A has also been widely used in the hybrid rice production [27]. Crossing the RILs with Zh9A has already resulted in the development of one commercial hybrid rice variety [34]. It is believable that QTLs detected in this study are helpful for designing an efficient molecular breeding strategy.

A total of 39 QTLs were identified for grain yield and its component traits in the RILs, testcross* F*_1_s, and H_MP_ across two years. Thirteen of them were common to the RILs and testcross* F*_1_s. In all cases the difference between TQ and IR24 and that between Zh9A/TQ and Zh9A/IR24 were in the same direction. This was in accordance with previous reports [5, 7, 9, 19, 20], providing the potential to increase the yield of hybrids by increasing the yield of parental lines. Comparison among QTLs detected from different datasets in our study also provides a chance to identify important genetic factors for heterosis. As shown in Table 1, a QTL detected in the RIL population has a different additive effect between TQ and IR24 alleles, and a QTL detected for H_MP_ has a different dominance effect between Zh9A/TQ and Zh9A/IR24 heterozygotes. Seventeen QTLs showed significant effects in the RIL population only, indicating that they have little dominance effect. Thus, they are QTLs with additive action mode (Table 5). Nine other QTLs were detected for either or both the testcross and H_MP_, indicating that they are overdominance QTLs having significant dominance effects and little additive effect. Included are* qNGP6.1*,* qNGP10*,* qNSP3.2*,* qSF5.2*,* qSF6*,* qTGW3.1*,* qTGW6*,* qGY5,* and* qGY10*. One more overdominance QTL is* qSF10* for which the effects estimated from testcrosses and H_MP_ were much higher than the value calculated from RILs. The remaining 12 QTLs had significant additive effects and appeared to have significant dominance effect; thus, they are QTLs with dominance action mode.

Most of the QTL detected in our study were clustery distributed in several chromosomal regions, including the intervals RM6–RM266 on chromosome 2, RM15139–RM16 on chromosome 3, RM437–RM18189 and RM274–RM334 on chromosome 5, RM469–RM190 and RM276–RM3330 on chromosome 6, RM70–RM18 on chromosome 7, and RM6704–RM228 on chromosome 10. All these regions have been found to harbor QTLs for yield traits in multiple studies [5, 7, 9, 19, 20, 25], suggesting that particular attention should be paid to these regions for marker-assisted improvement of rice yield potential in future studies.

It has been commonly observed that additive effects contribute a great proportion to variation of yield traits in segregating populations used for heterosis analysis [2, 5, 10, 13]. Similarly, we found that 13 of the 21 QTLs detected in the testcross population also showed significant effects in the RIL population. It has also been reported that dominance and overdominance play a critical role as a genetic basis of heterosis and heterotic loci did not usually overlap with QTLs for trait performance [3, 11, 15, 19, 23]. Indeed, eight QTLs detected in our testcross population were not found in the RIL population, including the QTL having the largest *R*^2^ for NGP,* qNGP10*, and the two QTLs for GY,* qGY5* and* qGY10*. Moreover, seven of the ten QTLs detected for H_MP_ in our study did not show significant additive effect.

It has been reported [4] that there may be two types of heterotic genes or QTLs. One is the gene showing an overdominance effect in a certain genetic background; the other is the gene showing no overdominance effect but providing a genetic background for the overdominance function of the type one gene. In the present study, two overdominance QTLs for GY were identified in RM437–RM18189 on chromosome 5 and RM6704–RM6100 on chromosome 10. The region RM6704–RM6100 also had a significant effect on NGP and SF which overlapped with the fertility restore gene* Rf1*/*Rf4* [35]. This region had no significant effective on GY in the RIL population. This result suggests that the fertility restoring gene* Rf1*/*Rf4* itself or genes tightly linked to it are important background factors for heterosis in rice. More studies using near isogenic lines could help to clarify the role of* Rf1/Rf4*.

## 5. Conclusion

A total of 39 QTLs for six yield traits were detected using trait data of a pair of RIL and its testcross populations and a set of H_MP_ data derived. Nineteen of them were common to different datasets and showed a consistent allelic direction, providing the potential to increase the yield of hybrids by increasing the yield of parental lines. Ten of the 39 QTLs were found to show overdominance action and 12 others acted as dominance QTLs. These results suggest that dominance and overdominance are the most important contributor to yield heterosis in rice, in which the accumulative effects of yield components play an important role.

## Figures and Tables

**Table 1:** Genetic effects of the QTL detected using datasets of the recombinant inbred line, testcross *F*_1_, and mid-parent heterosis.

Dataset	Effect^a^
Recombinant inbred line	*a*
Testcross *F*_1_	(*a*_2_ + *d*_2_)−(*a*_1_ + *d*_1_)
Mid-parent heterosis	*d*_2_ − *d*_1_

^a^ *a*, additive effect of replacing a Teqing allele with an IR24 allele; (*a*_2_ + *d*_2_)−(*a*_1_ +  *d*_1_), increase of the genetic effect when a Zhong 9A/Teqing heterozygote is replaced with a Zhong 9A/IR24 heterozygote; (*d*_2_ − *d*_1_), increase of the dominance effect when a Zhong 9A/Teqing heterozygote is replaced with a Zhong 9A/IR24 heterozygote.

**Table 2:** Mean trait values and standard deviation of the parent lines, reference *F*_1_s, and segregating populations.

Type	Name^a^	Trait^b^					
		NP	NGP	NSP	SF (%)	TGW (g)	GY (g)
Parent line	TQ	8.1 ± 2.1	200.4 ± 17.8	227.1 ± 30.2	88.5 ± 4.0	26.7 ± 0.0	33.1 ± 0.1
	IRBB52	11.2 ± 0.8	105.1 ± 33.1	130.1 ± 49.5	81.9 ± 5.6	25.5 ± 1.2	26.2 ± 4.3
	IRBB59	12.9 ± 1.8	100.0 ± 28.0	117.3 ± 42.0	86.6 ± 7.0	26.5 ± 0.2	30.2 ± 3.0
	Zh9B	8.6 ± 0.3	65.9 ± 26.6	107.7 ± 30.9	60.0 ± 7.6	22.3 ± 2.4	11.6 ± 6.0

Reference *F*_1_	Zh9A/TQ	10.6 ± 2.4	178.6 ± 56.7	225.5 ± 96.0	81.2 ± 9.5	26.5 ± 0.3	44.0 ± 0.9
	Zh9A/IRBB52	10.4 ± 2.3	131.6 ± 34.4	174.0 ± 59.7	76.7 ± 6.3	26.9 ± 0.9	31.7 ± 0.2
	Zh9A/IRBB59	12.8 ± 2.2	139.7 ± 15.2	175.4 ± 50.2	81.9 ± 14.5	27.8 ± 0.4	44.1 ± 6.8

Population	RIL	9.7 ± 1.1	137.1 ± 20.9	162.7 ± 26.0	85.4 ± 5.2	25.8 ± 2.7	29.5 ± 3.8
	TC	9.6 ± 1.2	140.1 ± 20.5	194.0 ± 18.7	73.1 ± 8.6	26.5 ± 1.6	30.8 ± 5.3

Derivative	H_MP_	0.5 ± 1.1	38.9 ± 17.5	59.2 ± 12.0	0.3 ± 7.9	2.5 ± 0.7	10.4 ± 4.9
	TC-RIL	−0.05	3.03	31.29^*∗∗*^	−12.38^*∗∗*^	0.72^*∗∗*^	1.33^*∗∗*^

^a^TQ, Teqing; Zh9B, Zhong 9B; Zh9A, Zhong 9A; RIL, recombinant inbred line; TC, testcross *F*_1_; *H*_MP_, mid-parent heterosis; TC-RIL, increase of trait value in TC over its corresponding RIL.

^b^Trait values are presented as mean ± sd. NP, number of panicles per plant; NGP, number of grains per panicle; NSP, number of spikelets per panicle; SF, spikelet fertility; TGW, 1000-grain weight; GY, grain yield per plant. ^*∗∗*^Significant at *P* < 0.01.

**Table 3:** Correlation coefficients between RILs, testcross *F*_1_s, and mid-parent heterosis.

Item^a^	Trait^b^					
	NP	NGP	NSP	SF	TGW	GY
RIL versus TC	0.375^*∗∗*^	0.446^*∗∗*^	0.675^*∗∗*^	0.406^*∗∗*^	0.902^*∗∗*^	0.377^*∗∗*^
RIL versus H_MP_	−0.103	−0.086	−0.022	0.110	0.200^*∗∗*^	0.014
TC versus H_MP_	0.882^*∗∗*^	0.840^*∗∗*^	0.684^*∗∗*^	0.953^*∗∗*^	0.598^*∗∗*^	0.925^*∗∗*^

^a^RIL, recombinant inbred line; TC, testcross *F*_1_; H_MP_, mid-parent heterosis.

^b^NP, number of panicles per plant; NGP, number of grains per panicle; NSP, number of spikelets per panicle; SF, spikelet fertility; TGW, 1000-grain weight; GY, grain yield per plant. ^*∗∗*^Significant at *P* < 0.01.

**Table 4:** Broad-sense heritability (%) of the six yield traits.

Populations^a^	Trait^b^					
	NP	NGP	NSP	SF	TGW	GY
RIL	54.53	30.82	26.64	54.92	94.50	45.93
TC	60.51	32.63	12.27	66.17	84.93	51.22

^a^RIL, recombinant inbred line; TC, testcross *F*_1_.

^b^NP, number of panicles per plant; NGP, number of grains per panicle; NSP, number of spikelets per panicle; SF, spikelet fertility; TGW, 1000-grain weight; GY, grain yield per plant.

**Table 5:** QTLs associated with yield traits detected for the phenotypic performance in the recombinant inbred line and testcross populations and for the mid-parent heterosis (H_MP_).

Trait^a^	QTL^b^	Interval	RIL		Testcross		H_MP_		Mode^e^
			Effect^c^	*R* ^2^ (%)^d^	Effect	*R* ^2^	Effect	*R* ^2^	
NP	*qNP2*	RM240-RM207	0.66	9.49	0.94	4.98			D
	*qNP9*	RM8206-RM219	0.35	4.06					A

NGP	*qNGP2*	RM240-RM207	−13.06	6.80	−12.17	2.11			D
	*qNGP3*	RM15303-RM16	−6.68	3.42 (0.68)	−10.32	1.83			D
	*qNGP4*	RM349-RM3333	4.62	1.51					A
	*qNGP5*	RM274-RM334	5.28	1.38					A
	*qNGP6.1*	RM190-RM587			7.31	0.52	8.30	2.31	OD
	*qNGP6.2*	RM549-RM3330	−4.95	1.09					A
	*qNGP7*	RM70-RM18	−5.54	1.39	−10.99	1.64			D
	*qNGP10*	RM6704-RM3773			18.88	7.39	22.31	23.01	OD

NSP	*qNSP2*	RM6-RM207	−14.06	7.50	−19.16	1.69			D
	*qNSP3.1*	RM15139-RM15303	−5.46	1.32					A
	*qNSP3.2*	RM15717-RM6759			−7.62	0.60	−7.19	2.99	OD
	*qNSP4*	RM6992-RM349	6.24	1.21					A
	*qNSP5*	RM274-RM334	6.04	1.10					A
	*qNSP6*	RM276-RM549	−7.47	1.50	−9.81	0.76			D
	*qNSP7*	RM70-RM18	−6.55	1.78					A
	*qNSP12*	RM511-RM28313	6.13	1.01	11.50	0.97			D

SF	*qSF1*	RM11869-RM12178	−1.62	3.31					A
	*qSF2*	RM6-RM240	1.39	3.63					A
	*qSF3*	RM15303-RM16	−1.28	3.84	−3.95	2.93			D
	*qSF5.1*	RM18038-RM18189	2.29	7.76 (1.21)					A
	*qSF5.2*	RM274-RM334					3.72	0.99	OD
	*qSF6*	RM190-RM587			4.46	2.20	3.71	1.28	OD
	*qSF8*	RM23001-RM210	1.38	3.68					A
	*qSF10*	RM6704-RM3123	1.04	1.93 (1.05)	10.88	27.21 (3.22)	10.09	22.81 (4.48)	OD
	*qSF12*	RM28313-RM28597	−2.06	3.69					A

TGW	*qTGW1*	RM11869-RM12178	0.58	4.44					A
	*qTGW2.1*	RM13576-RM263	0.54	5.65					A
	*qTGW2.2*	RM207-RM266	0.36	5.18	0.61	8.84			D
	*qTGW3.1*	RM14383-RM14629			−0.67	1.94			OD
	*qTGW3.2*	RM15139-RM16	1.45	27.21	2.33	37.83	0.84	6.65	D
	*qTGW5*	RM437-RM18189	−1.25	17.71	−0.90	6.16	0.41	1.48	D
	*qTGW6*	RM3330-RM7193			0.45	1.80			OD
	*qTGW10*	RM3123-RM228	−0.32	1.25	−0.51	1.98			D
	*qTGW12*	RM28597-RM17	0.55	3.79					A

GY	*qGY2*	RM6-RM240	−1.17	3.63 (1.54)					A
	*qGY5*	RM18038-RM18189			−3.89	4.12	−3.15	3.69	OD
	*qGY10*	RM6704-RM6100			3.55	4.87	3.09	5.11	OD

^a^NP, number of panicles per plant; NGP, number of grains per panicle; NSP, number of spikelets per panicle; SF, spikelet fertility; TGW, 1000-grain weight; GY, grain yield per plant.

^b^QTLs are designated following the rules recommended by McCouch and CGSNL [31].

^c^All the QTL effects were significant at the level of *P* < 0.01. In the RIL, the effect refers to the additive effect of replacing a Teqing allele with an IR24 allele. In the testcross, the effect refers to the increase of genetic effect when a Zhong 9A/Teqing heterozygote is replaced with a Zhong 9A/IR24 heterozygote. In H_MP_, the effect refers to the increase of dominance effect when a Zhong 9A/Teqing heterozygote is replaced with a Zhong 9A/IR24 heterozygote.

^d^Proportion of phenotypic variance explained by the given QTL. Value in parenthesis refers to the contribution due to genotype-by-environment interaction.

^e^Mode, genetic action mode. A, additive; D, dominance; OD, overdominance.

## References

[1] Yuan L.-P. (2014). Development of hybrid rice to ensure food security. *Rice Science*.

[2] Yu S. B., Li J. X., Xu C. G. (1997). Importance of epistasis as the genetic basis of heterosis in an elite rice hybrid. *Proceedings of the National Academy of Sciences of the United States of America*.

[3] Zhuang J.-Y., Fan Y.-Y., Wu J.-L. (2000). Identification of over-dominance QTL in hybrid rice combinations. *Hereditas*.

[4] Fan Y.-Y., Zhuang J.-Y., Li Q. (2001). Analysis of quantitative trait loci (QTL) for plant height and the relation between these QTL and QTL for yield traits in rice. *Acta Agronomica Sinica*.

[5] You A., Lu X., Jin H. (2006). Identification of quantitative trait loci across recombinant inbred lines and testcross populations for traits of agronomic importance in rice. *Genetics*.

[6] Chen S.-G., Shen X.-H., Cao L.-Y. (2010). QTL mapping for heterosis of yield traits in rice. *Scientia Agricultura Sinica*.

[7] Qu Z., Li L., Luo J. (2012). QTL mapping of combining ability and heterosis of agronomic traits in rice backcross recombinant inbred lines and hybrid crosses. *PLoS ONE*.

[8] Zhu D., Zhou G., Xu C., Zhang Q. (2016). Genetic components of heterosis for seedling traits in an elite rice hybrid analyzed using an immortalized F_2_ population. *Journal of Genetics and Genomics*.

[9] Xiang C., Zhang H., Wang H. (2016). Dissection of heterosis for yield and related traits using populations derived from introgression lines in rice. *The Crop Journal*.

[10] Xiao J., Li J., Yuan L., Tanksley S. D. (1995). Dominance is the major genetic basis of heterosis in rice as revealed by QTL analysis using molecular markers. *Genetics*.

[11] Li Z.-K., Luo L. J., Mei H. W. (2001). Overdominant epistatic loci are the primary genetic basis of inbreeding depression and heterosis in rice. I. Biomass and grain yield. *Genetics*.

[12] Abdelkhalik A. F., Shishido R., Nomura K., Ikehashi H. (2005). QTL-based analysis of heterosis for grain shape traits and seedling characteristics in an *indica-japonica* hybrid in rice (*Oryza sativa* L.). *Breeding Science*.

[13] Luo X., Fu Y., Zhang P. (2009). Additive and over-dominant effects resulting from epistatic loci are the primary genetic basis of heterosis in rice. *Journal of Integrative Plant Biology*.

[14] Bian J., Jiang L., Liu L. (2010). Identification of *japonica* chromosome segments associated with heterosis for yield in *Indica* × *japonica* rice hybrids. *Crop Science*.

[15] Xin X. Y., Wang W. X., Yang J. S., Luo X. J. (2011). Genetic analysis of heterotic loci detected in a cross between *indica* and *japonica* rice (*Oryza sativa* L.). *Breeding Science*.

[16] Chu S.-H., Jiang W., Lee J., Chin J. H., Koh H.-J. (2012). QTL analyses of heterosis for grain yield and yield-related traits in *indica-japonica* crosses of rice (*Oryza sativa* L.). *Genes & Genomics*.

[17] Zhang H., Jiang J.-H., Liu X.-L. (2013). QTL mapping and genetic analysis of eight outcrossing-related traits and its mid-parental heterosis in *Japonica* rice. *Chinese Journal of Rice Science*.

[18] Luo X.-J., Xin X.-Y., Yang J.-S. (2012). Genetic-basis analysis of heterotic loci in Dongxiang common wild rice (*Oryza rufipogon* Griff.). *Genetics Research*.

[19] Luo L. J., Li Z.-K., Mei H. W. (2001). Overdominant epistatic loci are the primary genetic basis of inbreeding depression and heterosis in rice. II. grain yield components. *Genetics*.

[20] Mei H. W., Luo L. J., Ying C. S. (2003). Gene actions of QTLs affecting several agronomic traits resolved in a recombinant inbred rice population and two testcross populations. *Theoretical and Applied Genetics*.

[21] Li L., He X., Zhang H. (2015). Genomewide mapping reveals a combination of different genetic effects causing the genetic basis of heterosis in two elite rice hybrids. *Journal of Genetics*.

[22] Hua J. P., Xing Y. Z., Xu C. G., Sun X. L., Yu S. B., Zhang Q. (2002). Genetic dissection of an elite rice hybrid revealed that heterozygotes are not always advantageous for performance. *Genetics*.

[23] Hua J., Xing Y., Wu W. (2003). Single-locus heterotic effects and dominance by dominance interactions can adequately explain the genetic basis of heterosis in an elite rice hybrid. *Proceedings of the National Academy of Sciences of the United States of America*.

[24] Shen G., Zhan W., Chen H., Xing Y. (2014). Dominance and epistasis are the main contributors to heterosis for plant height in rice. *Plant Science*.

[25] Zhou G., Chen Y., Yao W. (2012). Genetic composition of yield heterosis in an elite rice hybrid. *Proceedings of the National Academy of Sciences of the United States of America*.

[26] Huang X., Yang S., Gong J. (2015). Genomic analysis of hybrid rice varieties reveals numerous superior alleles that contribute to heterosis. *Nature Communications*.

[27] Sun Z., Zhi-Guo E., Wang L. (2014). Exploring assessment method of Chinese rice backbone parents. *Acta Agronomica Sinica*.

[28] Mei D.-Y., Zhu Y.-J., Yu Y.-H., Fan Y.-Y., Huang D.-R., Zhuang J.-Y. (2013). Quantitative trait loci for grain chalkiness and endosperm transparency detected in three recombinant inbred line populations of *indica*rice. *Journal of Integrative Agriculture*.

[29] SAS Institute (1999). *SAS/STAT User's Guide*.

[30] Yang J., Hu C., Hu H. (2008). QTLNetwork: mapping and visualizing genetic architecture of complex traits in experimental populations. *Bioinformatics*.

[31] McCouch S. R. (2008). Gene nomenclature system for rice. *Rice*.

[32] Xie F., He Z., Esguerra M. Q., Qiu F., Ramanathan V. (2014). Determination of heterotic groups for tropical *indica*hybrid rice germplasm. *Theoretical and Applied Genetics*.

[33] Tang S.-X., Wang X.-D., Liu X. (2012). Study on the renewed tendency and key backbone-parents of inbred rice varieties (*O. sativa* L.) in China. *Scientia Agricultura Sinica*.

[34] Zhuang J.-Y., Zhu Y.-J., Tu G.-Q. (2010). Gene pyramiding assisted breeding of hybrid rice combination Zhongyou 161 with high yield and high grain quality. *Hybrid Rice*.

[35] Wang Z., Zou Y., Li X. (2006). Cytoplasmic male sterility of rice with Boro II cytoplasm is caused by a cytotoxic peptide and is restored by two related PPR motif genes via distinct modes of mRNA silencing. *Plant Cell*.

